# Supplementary material for: Interpreting fMRI Studies in Populations with Cerebrovascular Risk: The Use of a Subject-Specific Hemodynamic Response Function
Source: Behav Sci (Basel). 2025 Oct 26;15(11):1457. doi: 10.3390/bs15111457 (PMC12649662; doi:10.3390/bs15111457)
Supplement: Supplementary file 1 [file behavsci-15-01457-s001.zip › behavsci-3824630-supplementary.pdf]

Supplementary Tables

Table S1. Age and Vascular Risk Effects on Encoding-Related Brain Activity using the Canonical HRF

MNI Coordinates			Region	BA	T-value	Cluster size (voxels)
x	y	z				
Canonical HRF - Factor 1						
Positive Age Effect						
64	-26	42	R Supramarginal Gyrus	2	5.82	334
-60	-26	32	L Supramarginal Gyrus	40	4.33	124
Negative Age Effect						
-20	0	28	L Caudate	-	5.25	137
-2	-34	62	L Paracentral Lobule	6	4.56	142
Positive Vascular Risk Effect						
No significant clusters						
Negative Vascular Risk Effect						
No significant clusters						
Positive Age x Vascular Risk Effect						
No significant clusters						
Negative Age x Vascular Risk Effect						
-28	-60	22	L Middle Temporal Gyrus	39	6.05	107
Canonical HRF - Factor 2						
Positive Age Effect						
64	-26	42	R Supramarginal Gyrus	3/2	5.59	208
-60	-26	32	L Supramarginal Gyrus	40	4.31	107
Negative Age Effect						
No significant clusters						

Positive Vascular Risk Effect						
No significant clusters						
Negative Vascular Risk Effect						
No significant clusters						
Positive Age x Vascular Risk Effect						
No significant clusters						
Negative Age x Vascular Risk Effect						
No significant clusters						
Canonical HRF - Factor 3						
Positive Age Effect						
No significant clusters						
Negative Age Effect						
No significant clusters						
Positive Vascular Risk Effect						
No significant clusters						
Negative Vascular Risk Effect						
No significant clusters						
Positive Age x Vascular Risk Effect						
16	36	20	R Anterior Cingulate Cortex	32	4.49	129
0	32	8	R Anterior Cingulate Cortex	32	4.25	121
Negative Age x Vascular Risk Effect						
No significant clusters						

Table S2. Age and Vascular Risk Effects on Encoding-Related Brain Activity using the Subject-specific HRF

MNI Coordinates			Region	BA	T-value	Cluster size (voxels)
x	y	z				
Subject-specific HRF - Factor 1						
Positive Age Effect						
64	-26	44	R Supramarginal Gyrus	2	5.23	327
-58	-26	32	L Supramarginal Gyrus	40	4.75	151
Negative Age Effect						
-16	-48	22	L Cingulate Cortex	31	5.14	102
-2	-34	62	L Paracentral Lobule	6	5.58	623
Positive Vascular Risk Effect						
No significant clusters						
Negative Vascular Risk Effect						
No significant clusters						
Positive Age x Vascular Risk Effect						
No significant clusters						
Negative Age x Vascular Risk Effect						
-28	-60	22	L Middle Temporal Gyrus	39	6.65	184
-18	24	20	L Caudate	-	5.20	117
-34	-64	-48	L Cerebellum	-	4.08	119
Subject-specific HRF - Factor 2						
Positive Age Effect						
66	-26	42	R Supramarginal Gyrus	3/2	5.08	187
-58	-26	30	L Supramarginal Gyrus	40	4.82	149
Negative Age Effect						
0	-28	72	L Paracentral Lobule	6	6.32	512

Positive Vascular Risk Effect						
No significant clusters						
Negative Vascular Risk Effect						
No significant clusters						
Positive Age x Vascular Risk Effect						
No significant clusters						
Negative Age x Vascular Risk Effect						
-24	-60	20	L Cuneus	-	5.22	119
Subject-specific HRF - Factor 3						
Positive Age Effect						
No significant clusters						
Negative Age Effect						
6	-24	74	R Paracentral Lobule	6	4.60	169
Positive Vascular Risk Effect						
No significant clusters						
Negative Vascular Risk Effect						
No significant clusters						
Positive Age x Vascular Risk Effect						
No significant clusters						
Negative Age x Vascular Risk Effect						
No significant clusters						

Figure S1. Age Decreases in Encoding Activity as a Function of HRF Type Controlling for Vascular Factor 1.

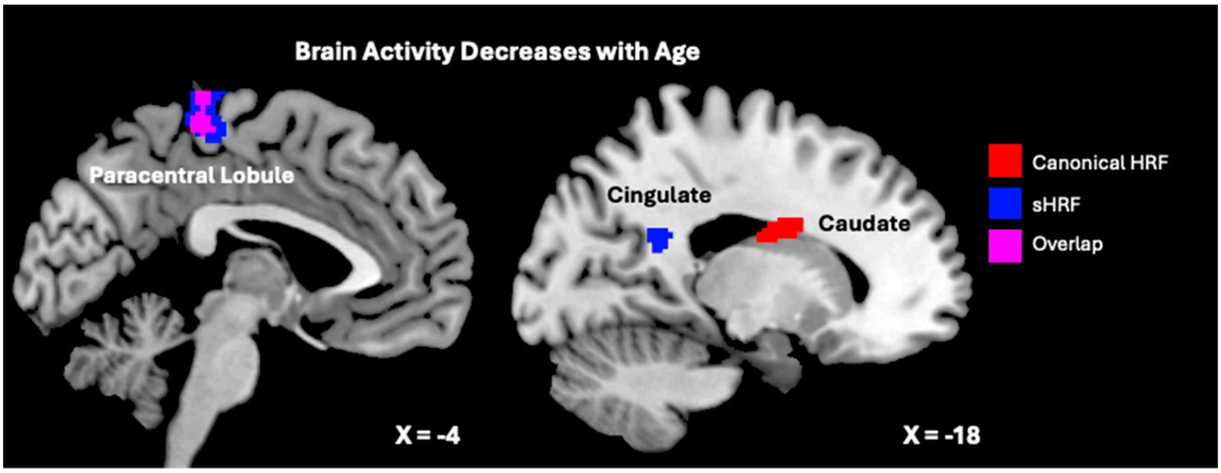

Both HRF types show overlapping relationships with age in the paracentral lobule (pink), but differ in their relationships with age in the cingulate cortex (blue: sHRF) and caudate (red = caudate).

Figure S2. Interaction Between Vascular Factor 1 and Age as a Function of HRF Type

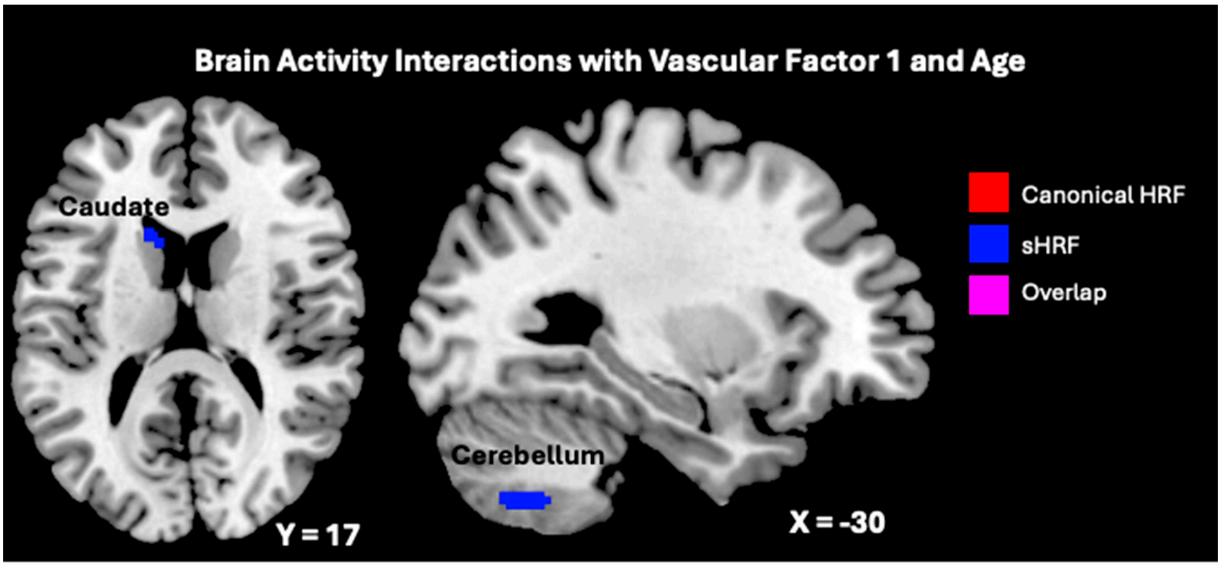

Only the sHRF revealed interactions between age and vascular factor 1 in the caudate and cerebellum (blue: sHRF).

Figure S3. Interaction Between Vascular Factor 2 and Age as a Function of HRF Type

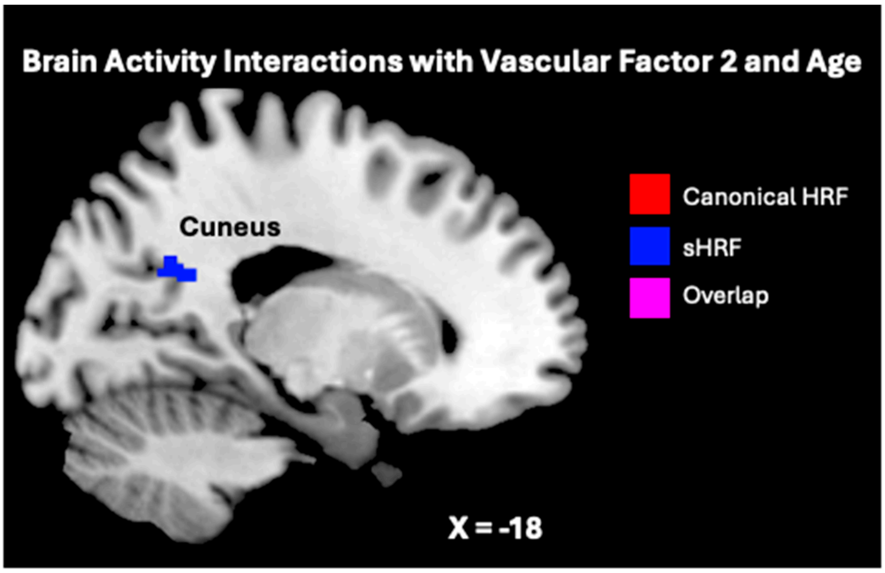

Only the sHRF revealed interactions between age and vascular factor 2 in the cuneus (blue).

Figure S4. Interaction Between Vascular Factor 3 and Age as a Function of HRF Type

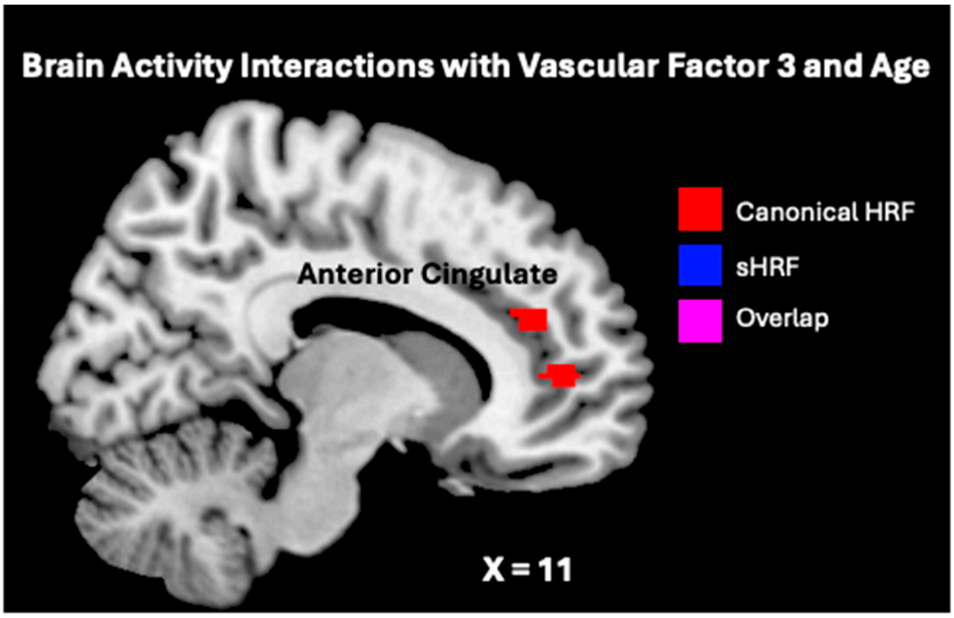

Only the sHRF revealed interactions between age and vascular factor 3 in the anterior cingulate (red).
